# Molecular Simulation of Pervaporation on Polyurethane Membranes

**DOI:** 10.3390/membranes13020128

**Published:** 2023-01-19

**Authors:** Ivan P. Anashkin, Alexander V. Klinov, Ilsiya M. Davletbaeva

**Affiliations:** 1 Department of Chemical Process Engineering, Kazan National Research Technological University, 420015 Kazan, Russia; 2 Department of Synthetic Rubber, Kazan National Research Technological University, 420015 Kazan, Russia

**Keywords:** molecular dynamics, pervaporation, polyurethane, amino ether of boric acid, water, ethanol

## Abstract

This article discusses a molecular simulation of membrane processes for the separation of liquid mixtures during pervaporation. A method for simulating the structure of polyurethane membranes was developed. The method was based on the known mechanisms of the formation of macromolecules from constituent monomers. For the formation of a chemical bond between the monomers, values of the parameters of the potentials of intermolecular interactions were set so that bonds were formed only between the corresponding atoms. The algorithm was validated to produce polymer films from diphenylmethane diisocyanate (MDI) and amino ethers of boric acid (AEBA). The polymer film obtained according to the developed algorithm was used to study the adsorption of ethanol and water. The concentration distributions of the components inside the polymer film were obtained for films of various thicknesses. Modifications of the DCV-GCMD method were proposed for the molecular simulation of pervaporation. The algorithm was based on maintaining a constant density of the mixture in the control volume. After the molecules were added to the control volume, thermodynamic equilibrium was established. During this process, molecules moved only in the control volume, while the rest of the molecules were fixed. The proposed algorithm was used to calculate the flows of water and ethanol through the polymer film.

## 1. Introduction

Liquid mixture separation processes are part of many industrial technologies. Often, these processes are associated with large expenditures of energy and material resources. For example, in distillation processes, the separated mixture is evaporated, which requires a significant amount of heat. In extraction processes, it is not easy to select an extractant, the cost of which can be high, but its regeneration is again carried out mainly by the rectification method. Adsorption processes are characterized by the extraction of substances at low concentrations and there is need to solve the problem of adsorbent regeneration. Membrane technologies overcome many shortcomings of liquid mixture separation processes traditionally used in industrial technology. The main advantages of membrane separation processes for both gas and liquid mixtures are high selectivity, low waste, and ease of scaling. One of the membrane processes for separating liquid mixtures is pervaporation [1,2].

In the process of pervaporation, separation of the liquid mixture occurs using diffusion membranes due to the created difference in partial pressures: a vacuum is created in the permeate region, and atmospheric or slightly higher pressure is maintained in the raw material. The success of the practical application of pervaporation for the separation of certain liquid mixtures depends on the separation characteristics of the selective membrane layer. The most important of these characteristics are flux and selectivity. The choice of material for the selective layer of the membrane is not an easy task due to the large number of currently known and newly synthesized materials. Conventionally, the materials used to create a selective layer of pervaporation membranes can be divided into organic (polymer), inorganic (ceramics, metal, graphene, etc.), and hybrid organic-inorganic (organometallic, ceramic-organic, etc.) materials.

A promising method for assessing the separation characteristics of a material for a selective layer of membranes is molecular dynamics (MD) [3]. This method makes it possible to simulate equilibrium and non-equilibrium states in multiphase, multicomponent systems based on the intermolecular interactions of substances included in the system under consideration. MD simulation describes the trajectories of the molecules of the system under consideration, which underlie the phenomena occurring in the process of separation of a liquid mixture by a pervaporation membrane. As is known, the process of pervaporation can be represented as the stages of selective adsorption (dissolution) of the components of the mixture in the membrane and long-range diffusion of the components through the membrane [4]. MD modeling can be performed for these stages separately under equilibrium conditions by determining the sorption isotherm and Einstein diffusion coefficients [5]. These data are further used in mass transfer macromodels (Langmuir sorption isotherm equation, Fick diffusion equation) to calculate the membrane separation characteristics. The combination of methods for determining the adsorption isotherm and calculating the diffusion coefficients makes it possible to replace a physical experiment with a numerical one. For example, in ref. [6], the process of pervaporation of an ethanol–water mixture on a silicolite membrane was studied. Molecular methods were used to simulate the processes of adsorption and diffusion of pure components. The results of the numerical experiments were used to adjust the parameters of the adsorption and diffusion models. The resulting models were used to calculate the fluxes of components. The results obtained for ethanol were in good agreement with the experimental data. The calculated water flows exceeded the experimental values by a factor of two. The authors attributed this discrepancy to the inaccurate determination of membrane thickness in the physical experiment. In ref. [7], a similar approach was used to study the separation of water–alcohol mixtures on membranes with a selective layer of zeolite decadodecasil 3R [8].

There are also MD simulation methods for the simultaneous description of all stages of the membrane separation process under nonequilibrium conditions. Many algorithms have been proposed for the molecular study of different variations of membrane separation. In these methods, the membrane divides the simulation box into two regions in which the specified conditions are maintained. For example, one common method is DCV-GCMD [9,10]. This method is based on the allocation of control volumes in two regions. In these volumes, the chemical potential is maintained constant by inserting and removing particles to the appropriate control volumes. In ref. [11,12], this algorithm was modified and the density of the component was maintained constant in the control volumes. This made it possible to achieve high densities in the control volumes and simulate the process of pervaporation.

The authors of ref. [13] proposed an algorithm for simulation of the pervaporation process. According to the proposed algorithm, the liquid phase was in equilibrium with the vapor, and all molecules were removed from the gas phase region. The vapor and gas phases were separated by a rigidly fixed layer of graphene. Permeability was determined through the pressure difference in the vapor in equilibrium with the liquid phase and the permeate. A similar algorithm was used to study the separation of an ethanol–water mixture [14,15,16] by various membranes.

There are other approaches to keep the driving force of the separation process in constant. In ref. [17], an algorithm was proposed to maintain a constant pressure drop. Using the developed method, the process of diffusion of the Lennard–Jones fluid through cylindrical pores was studied. The essence of the method was that one of the surfaces of the simulated box was a wall with repulsion. By moving this surface, a constant pressure on the feed side mixture was achieved.

Recently, polymeric materials were actively used to create selective layers of pervaporation membranes [18], which has wide possibilities for modern methods of polymer synthesis. Molecular dynamics methods are widely used for simulation of polymer systems [19,20], including simulation of polyurethanes [21,22,23].

In this paper, we propose methods for simulation of the molecular structure of polymeric membranes and the process of pervaporation on these membranes. The proposed methods and programs were tested on the study of adsorption and mass transfer through the membrane in an ethanol–water system. 

## 2. Methods

### 2.1. Molecular Interaction

**Figure 1:**
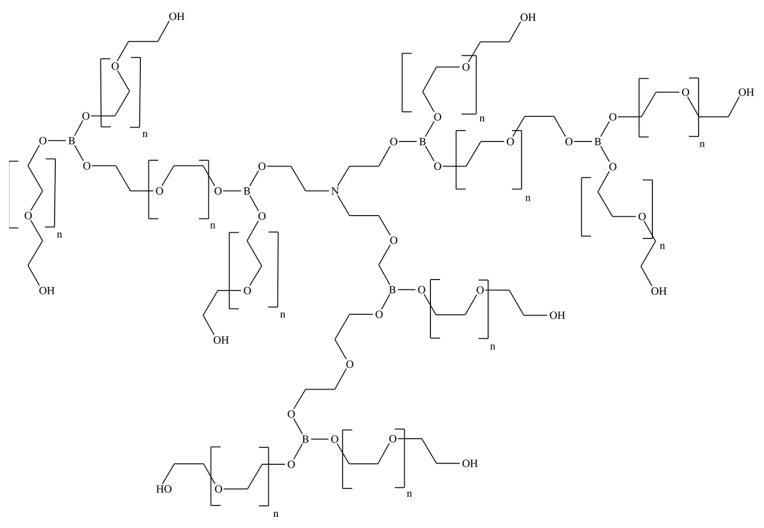
Chemical structure of the AEBA molecule.

**Figure 2:**
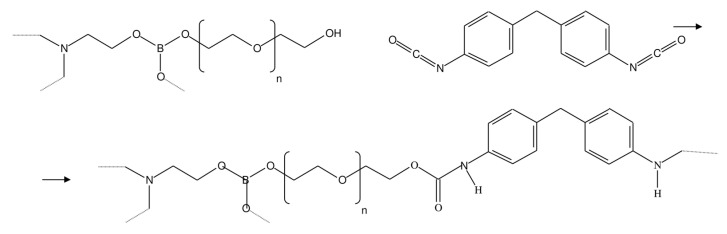
Crosslinking reaction of AEBA and MDI molecules.

In this work, pervaporation membranes based on polyurethane synthesized from amino ethers of boric acid (AEBA) were studied. These polyurethane membranes were obtained by crosslinking AEBA and diphenylmethane diisocyanate (MDI). Figure 1 shows AEBA from which the polyurethanes were synthesized [24,25]. The AEBA molecule was synthesized from triethanolamine, boric acid, and polyethylene glycol in a molar ratio of 1:6:12. The AEBA molecule is relatively large, has a polyol chain length of n = 8, and has 6 terminal OH groups at which the polymerization reaction could proceed [26]. During polymerization (Figure 2), crosslinking can occur both between different AEBA molecules and within one AEBA molecule.

A combination of different force fields was used to describe the intermolecular interactions of AEBA in the synthesized polyurethane. The parameters from ref. [23] were used to describe the MDI interaction, and the TraPPE force field [27,28,29,30] was used to describe the interaction of the AEBA part of polymer. The parameters of the intermolecular interactions of the boron atoms were taken from a previous study [31].

The force field TIP4P/2005 [32] was used to describe the intermolecular interaction of water. For ethanol, the TraPPE force field [27] was used. The combination of these two force fields enabled the description of the Einstein diffusion coefficients in an ethanol–water mixture with high accuracy [33]. By default, the cross parameters were determined by the Lorentz–Berthelot mixing rule.

The parameters of the intermolecular interactions are presented in the Supplementary Materials.

### 2.2. Method for Modeling the Structure of Polyurethanes

An important step in the simulation was the creation of the molecular structure of the membrane under study. Well-known software systems such as Assemble! [34] are not suitable for modeling the structure of polyurethanes, as they have a complex branched three-dimensional structure. Usually, the structures generated by such programs have high energy, so further relaxation of the polymer chain is a big problem in the generation of the membrane structure [35].

To solve this problem, an algorithm for generating the molecular structure of a polyurethane membrane based on quasi-chemical reactions was developed. A similar approach was used in ref. [36].

**Figure 3:**
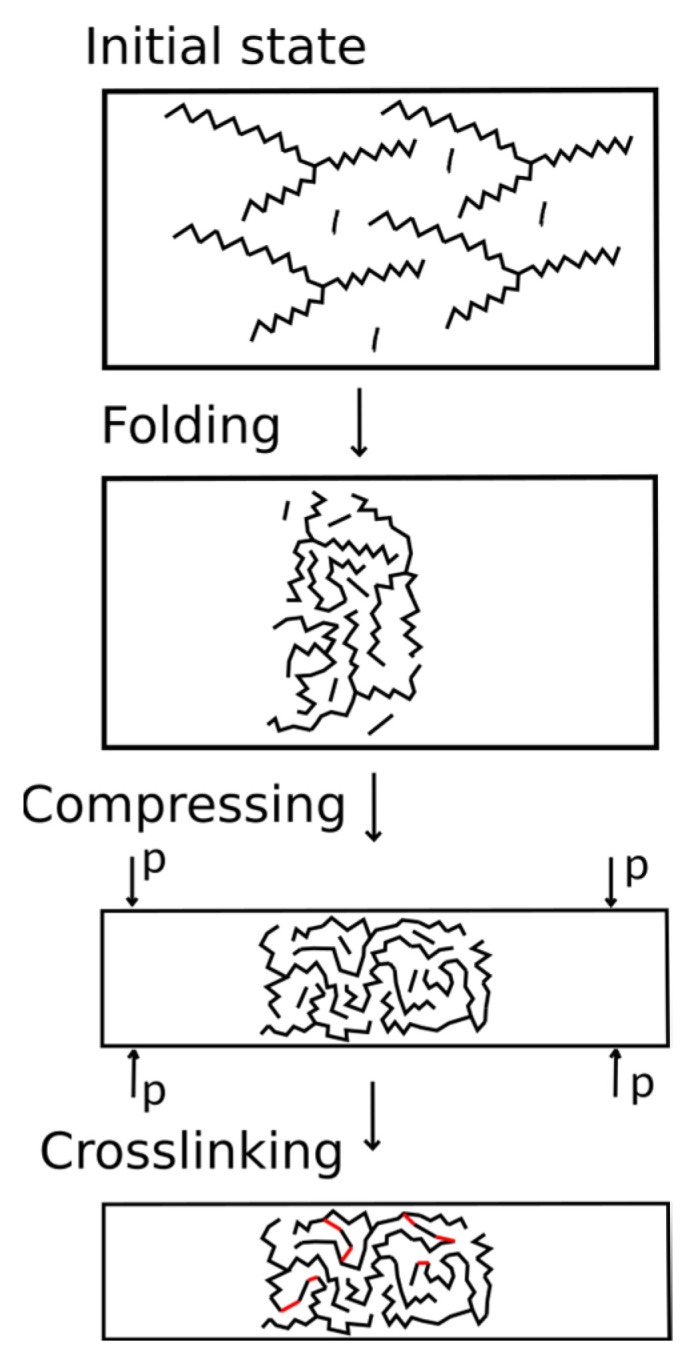
Algorithm for creating the structure of polymers.

The developed algorithm simulated the initial mixture of monomers with a given composition. The scheme of the algorithm is shown in Figure 3. During the first stage, the AEBA and MDI molecules were randomly placed in a large simulation box using the packmol package [37,38]. The box size was determined by the number of added molecules since the AEBA molecule is relatively large and achieve a dense placement of this molecule is problematic. The box size along the z axis was 3 times larger than that along the x and y axes, in order for folding to expand along the x and y axes. The polymer formed a phase interface oriented along the normal direction to the z axis.

During the second stage, MD simulation of the system in the box was carried out. This stage was carried out at a constant volume and a given temperature (NVT ensemble). At this stage, AEBAs with long tails “mix” with each other and with MDI molecules. As a result, the system is compacted.

During the third stage, the simulation was carried out in the NPT ensemble. It is important to note that the anisotropic Parrinello–Rahman algorithm was used. The compressibility along the x and y axes significantly exceeded the compressibility along the z axis. The value of the reference pressure was controlled by the dimensions of the membrane along the axes. We chose a pressure value so that the x and y dimensions of the system would be greater than 4 nm.

During the third stage, crosslinking was simulated in the NVT ensemble. During chemical interaction, the reacting sections of the molecules must move close and have sufficient energy for an elementary reaction to occur. However, due to low diffusion coefficients, complete reaction between the components can take a significant time. Therefore, in this work, we artificially accelerated the approach of the reacting groups by replacing the cross parameters.

**Figure 4:**
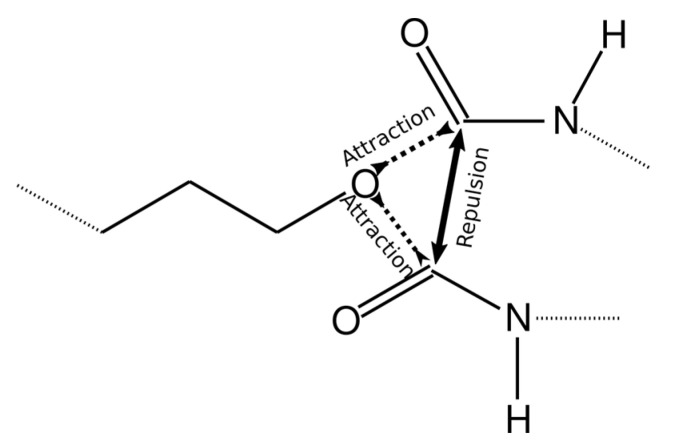
Scheme describing intermolecular interactions to achieve the formation of only one chemical bond.

To achieve this, the value of the interaction energy ε_CO_/k_B_ between the reacting groups (O atom in the AEBA molecule and C atom in MDI) was increased to 6000 K. However, in this case, more than one MDI molecule can come close to one O atom (Figure 4) or more than one AEBA molecule to the C atom. Therefore, more than one chemical bond can be formed between these atoms. To exclude bonding between identical groups, the values of σ_OO_ and σ_СС_ were increased to 0.6 nm, which did not allow identical groups to move close.

The Lennard–Jones potential decays strongly with increasing distance. Therefore, if the values of the cross parameter σ_OC_ are immediately set equal to the bond length, then the oxygen atoms in AEBA and carbon atoms in MDI will weakly interact and their convergence will take a long time. Therefore, the values of σ_OC_ gradually decreased from 0.6 nm to values equal to the bond length of 0.14 nm.

After the MD simulation, the oxygen and carbon atoms located at a distance of less than 1.7 nm were determined. Subsequently, the topology of the molecules changed and a bond was formed between these atoms. Additionally, new bonds and angles were written to the topology file. The resulting structure and topology of the polyurethane molecules were subsequently used to study adsorption and membrane separation.

### 2.3. Method for Simulation of Pervaporation on Polymeric Membranes

A program in ref. [39] was developed for MD simulation of the perforation process. The simulation method was based on the use of the dual control volume approach (DCV- GCMD).

**Figure 5:**
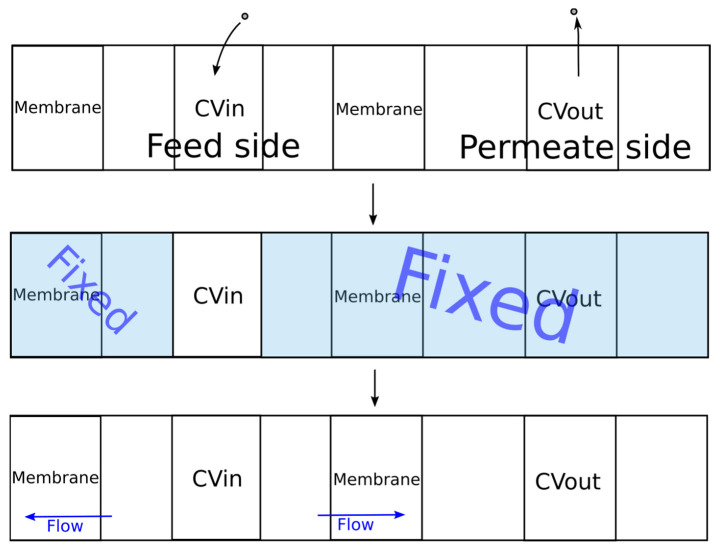
The developed algorithm for simulation of the pervaporation process.

The original DCV-GCMD method was based on maintaining a constant value of the chemical potential in the control volumes, which limited its scope for dense systems (liquid mixtures). To solve this problem, the algorithm shown in Figure 5 was proposed.

During the first stage, molecules were added to the feed control volume (CVin) using the packmol program until the specified density was reached. Then, molecules from the control volume of the permeate (CVout) were removed until the required pressure value was reached, which was calculated using the equation of state of an ideal gas. Next, MD simulation was carried out using the gromacs package [40,41,42] to establish thermodynamic equilibrium in the control volume.

During the second stage of simulation, the coordinates of all molecules outside the control volume CVin were fixed. The specified temperature was maintained only for the molecules in the control volume. When simulating a liquid phase, it is rather difficult to find free space for inserting new molecules in order to maintain a given density. In this regard, the energy of the system can significantly increase after insertion of a molecule due to the overlapping of molecules, leading to inaccuracies in the simulation or stopping of the calculation. To solve this problem, all values of the parameter ε were multiplied by a coefficient. Several successive calculations were carried out in which the value of the coefficient was changed from 0.01 to 1.0 according to the exponential law from the simulation iteration number. Thus, smooth insertion of the molecules was provided. The simulation time at each such iteration was 1 ps. Next, to establish thermodynamic equilibrium inside and maintain the set temperature, the dynamics of the molecules were simulated for 20 ps. The integration step of the equation of motion was 0.001 ps.

During the third stage, the molecules outside the control volume were assigned the same velocities as before the second stage. The simulation of the dynamics of the molecules was already carried out for the entire system, which corresponded to the process of pervaporation. In order to prevent the membrane from moving under the pressure of the molecules of the liquid phase, the membrane molecules were fixed. The developed program implemented two types of membrane fixation. For crystalline membranes, in which the molecular coordinates were inactive, fixation of all membrane molecules can be used. In the case of a polymeric membrane, swelling is observed and the structure of the membrane changes. In this regard, for adequate simulation of the polymer membrane, it was necessary to ensure the mobility of the polymer chain. To implement this possibility, a certain number of atoms in the membrane were set, the coordinates of which were fixed.

A Nose–Hoover thermostat was used to maintain a constant temperature in the system. Periodic boundary conditions for all coordinates were used. The simulation time for molecular dynamics during the third stage was 100 ps. Subsequently, the energy values of the system were calculated, and the distribution values of the mixture densities and the membrane along the z axis were averaged. The flux of components was determined by the number of component molecules that passed over the surface area over a period of time:

(1) $$j_{A}=\frac{N_{A}}{S\Delta t},$$

where *N* is the number of molecules of component *A* passed through the membrane, *S* is the area of the membrane, and Δ*t* is the time for which the molecules passed.

After that, the cycle was repeated.

During the initial stage, adsorption and an increase in the concentration of components on the membrane occurred. Therefore, the averaging of the fluxes and distributions of the component densities was carried out only after the establishment of stationarity.

The dimensions of the simulated system along the x and y axes corresponded to the size of the membrane. The length of the feed side was 12 nm, the length of the control volume Cvin was 6 nm, the permeate side was 40 nm, and Cvout was 10 nm.

### 2.4. Simulation of Adsorption under Equilibrium Conditions

The simulation of adsorption was carried out by adding 7000 thousand atoms to the free space of the box. Semi-isotropic pressure maintenance conditions were used, the compressibility of the system was equal to zero along the x and y axes, and the pressure value was smoothly set to 1 bar along the z axis. Simulation was carried out for 2 ns at a given pressure at which thermodynamic equilibrium was established. Next, the volume of the system was fixed and a 5 ns simulation was carried out, in which the density distribution of the components along the z axis was determined.

## 3. Results and Discussion

For the molecular simulation, a sample membrane based on AEBA was used. This sample membrane was obtained from 10 AEBA atoms and 40 MDI atoms. After the polymer crosslinking procedure, the dimensions of the membrane layer were 6.175 nm along the x and y axes and approximately 2 nm along the z axis. The smaller thickness of the obtained membrane sample was due to the need for a large cross-sectional area of the membrane to increase the flow. The analysis showed that out of 80 possible chemical bonds, 79 were formed. The density of the resulting membrane sample in a vacuum was 1350 kg/m^3^. For polymers synthesized from AEBA and polyisocyanate (product mixture of polymethylenepolyphenylene isocyanate and MDI), the density is 1143 kg/m^3^ [43]. Although a direct comparison was not valid in this case, AEBA–MDI polyurethane had a higher density due to the shorter chain length.

### 3.1. Adsorption of Water and Ethanol on Polyurethanes from AEBA

**Figure 6:**
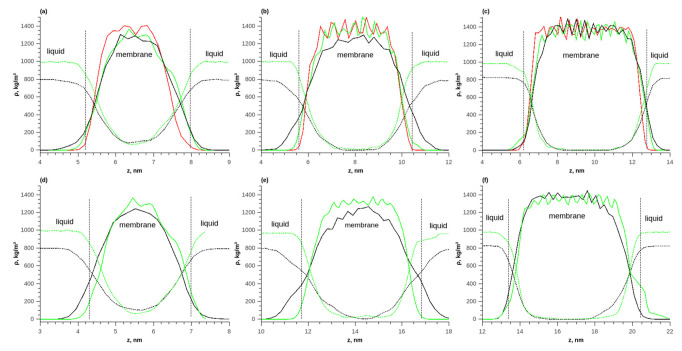
The density distributions of components along the length of the simulated box during the adsorption of pure water and ethanol on the membrane; (**a**) T = 298 K, 1 layer; (**b**) T = 298 K, 2 layers; (**c**) T = 298 K, 3 layers; (**d**) T = 353 K, 1 layer; (**e**) T = 353 K, 2 layers; (**f**) T = 353 K, 3 layers. Solid red line—polymer density in vacuum, solid green line—polymer density during water adsorption, solid black line—polymer density during ethanol adsorption, dotted green line—water density, and dotted black line—ethanol density.

The obtained sample was used to study the adsorption of water and ethanol in the membrane. Three variants with different numbers of membrane layers (from one to three) were investigated. The layers were initially located at a minimum distance from each other. During simulation in a vacuum, the layers stuck together due to intermolecular attraction. Additionally, no changes in density were observed at the junctions of the layers. Figure 6 shows a comparison of the density distributions of the membrane in a vacuum, as well as during the adsorption of pure water and ethanol at temperatures of 298 and 353 K. It can be seen that during the adsorption of water and ethanol in the membrane, swelling occurred because of the penetration of water and ethanol molecules into the polymer.

With a membrane thickness of 1 layer, the concentration constantly changed inside the membrane and there was no plateau corresponding to an equilibrium concentration of the solute in the polymer. For a membrane of 3 layers, such a plateau in the density distribution was observed. The thickness of the transition layer was approximately 2 nm, which corresponded to the thickness of one polymer layer. As the temperature increased from 298 to 353 K, the equilibrium density of water and ethanol in the membrane increased.

**Figure 7:**
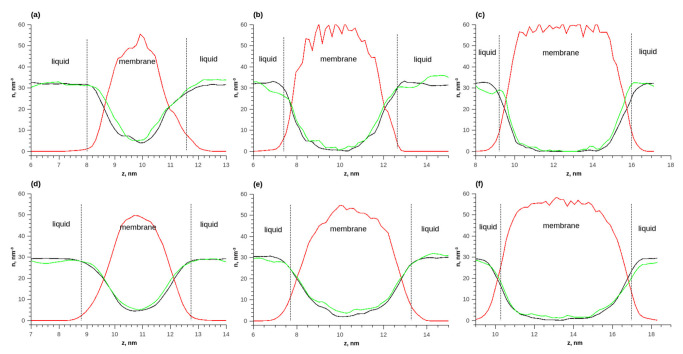
The density distributions of components along the length of the simulated cell during the adsorption of ethanol–water equimolar mixture on the membrane; (**a**) T = 298 K, 1 layer; (**b**) T = 298 K, 2 layers; (**c**) T = 298 K, 3 layers; (**d**) T = 353 K, 1 layer; (**e**) T = 353 K, 2 layers; (**f**) T = 353 K, 3 layers. Solid red line—polymer density in vacuum, solid green line—polymer density during water adsorption, solid red line—polymer density, solid green line—water density, and solid black line—ethanol density.

Figure 7 shows the distributions of numerical densities at different temperatures and quantities of membrane layers for the adsorption of an ethanol–water mixture. Due to the fact that the molar ratio of water and ethanol was 1 to 1, it was more convenient to analyze the distributions of numerical densities (the number of molecules per unit volume). As the membrane thickness increased, the number densities of ethanol and water in the membrane increased. The density of water in the membrane was greater than that of ethanol. Additionally, with an increase in the membrane thickness, the ratio of the density of water to the density of ethanol increased. Thus, the membrane adsorbed water better.

### 3.2. Pervaporation of Water and Ethanol on the Polyurethane Membrane

The mass transfer of an equimolar mixture of ethanol–water was studied on a prepared sample of the membrane film. To achieve this, the density of this mixture in the NPT ensemble was determined. The simulated system contained a total of 2000 molecules. A Parrinello–Rahman barostat was used and the reference pressure was 1 bar. As a result of simulation, the number density of water and ethanol was 7.6 nm^−3^. This density value was maintained in the control volume Cvin. The pressure in the control permeate volume was assumed to be zero (all molecules were removed from CVout). The simulation temperature was 353 K; a Nose–Hoover thermostat was used. The simulation lasted approximately 300 cycles, one third of which was devoted to the establishment of stationarity. Next, the average value of the flow of components over cycles was determined. For a two-component mixture, the separation coefficient was determined by the expression [44]:

(2) Sw=xwPxePxwFxeF,

where the indices *x* are the mole fraction of the component, the subscripts correspond to *w*—water, *e*—ethanol, and the superscripts correspond to *f*—feed side, *p*—permeate side. For our case $x_{w}^{F}=x_{e}^{F}=0.5$, and the molar fraction of the components could be calculated from the molar flow xwPxeP=jwje, so Sw=jwje.

The permeability coefficient was determined by the expression:

(3) $$P_{w}=\frac{j_{w}\delta }{p_{w}^{F}-p_{w}^{P}}\;,$$

where *δ* is the membrane thickness, and *p* is the partial pressure of the mixture component. This value was determined from the expression $p_{w}=p_{w}^{0}x_{w}\gamma_{w}$. The vapor pressure of the pure component $p_{w}^{0}$ was determined by CoolProp package [45], and the gamma activity coefficients were determined from the NRTL model.

The simulation results are presented in Table 1. The component fluxes exceeded the values for real membranes used in the industry by 5 orders of magnitude. This was due to the fact that the simulated membrane had a thickness of only 2–4 nm, while the thickness of the selective polymer layers of real membranes usually ranges from 1 to 100 μm. Analysis of the component flows showed that the ethanol flow exceeded the water flow. This was also due to the small thickness of the membrane.

According to the experimental data, the studied membranes synthesized from AEBA should have water selectivity [46], which contradicted the results obtained.

Ethanol, when mixed with water in 50% molar volume, is highly volatile and, therefore, the driving force of the process of its mass transfer will be greater. In the absence of membrane resistance to mass transfer, the separation coefficient would be determined by the composition of the vapor phase with a given composition and temperature ($x_{w}^{F}$ = 0.5, T = 353K) and would be equal to the relative volatility $S_{w}=\frac{p_{w}^{0}x_{w}\gamma_{w}}{p_{e}^{0}x_{e}\gamma_{e}}=0.525$.

When considering flows through a membrane film with a thickness of 1 layer, the value of $S_{w}$ = 0.522 practically did not differ from the coefficient of relative volatility of the components, which characterized their equilibrium distribution between liquid and vapor during open evaporation. In this case, almost the entire membrane was a boundary layer. Additionally, the ethanol concentration in the membrane was much higher compared to that in thicker membranes. Thus, the difference in the adsorption of the components on the membrane did not make a significant contribution to the separation of the components. Moreover, in view of the insufficient thickness of the membrane, the difference in the diffusion coefficients of the components would not significantly affect the distribution of concentrations.

With an increase in the thickness of the membrane layer, a more than 2-fold decrease in the flow was observed. An increase in the $S_{w}$ coefficient was also observed and the permeability coefficient of water increased, while that of ethanol decreased.

Thus, when studying polymeric membranes using the MD method, it is necessary to study the effect of membrane size on the permeability and separation coefficients. These characteristics should asymptotically approach constant values with increasing membrane thickness, since the contribution of the boundary layer will become negligibly small with increasing membrane thickness. These phenomena require further study.

## 4. Conclusions

In this work, an algorithm for constructing the molecular structure of a polymer membrane from its constituent monomers was developed. The algorithm consisted of several stages and led to the formation of a branched polymer structure. The polymer film was constructed from polyurethane based on AEBA. Comparison of the density of the molecular model of the polymer with its close physical counterpart showed a discrepancy of 18%.

The resulting molecular structure of the polymeric polyurethane film was used as a pervaporation membrane in the separation of an ethanol–water mixture. The adsorption characteristics of the components of this mixture on the membrane and the separation characteristics were studied, in the form of flows and selectivity coefficients. To study the separation characteristics of the polymer membrane, an algorithm and program for MD simulation of the pervaporation process under nonequilibrium conditions were developed. The disadvantage of the proposed method includes that it was difficult to determine the pressure in the control volume CVin of the liquid phase. The pressure was actually determined by indirect calculation during simulation under the same conditions.

The results of the adsorption simulation showed that there were no adsorption layers at the membrane–liquid phase interface and the corresponding peaks on the single-particle density distribution function. A monotonous change in the density of the sorbed substance in the liquid phase to that at equilibrium in the membrane occurred in the transition layer of approximately 2 nm thick. With an increase in temperature from 298 to 353 K, an increase in the equilibrium density of water and ethanol in the membrane was observed.

MD simulation of the pervaporation process was carried out for an equimolar mixture of ethanol–water for membranes with thicknesses of 2 and 4 nm. It was shown that for a membrane with a thickness of 2 nm, the value of the separation coefficient for water turned out to be close to the coefficient of the relative volatility of water in the mixture being separated. This was due to the weak influence of a membrane of such thickness on the mixture separation process as a result of the transition of its components from liquid to vapor due to significant swelling. With an increase in the membrane thickness to 4 nm, an increase in the separation coefficient for water by a factor of 1.5 was observed, which characterized the membrane under study as selective to water.

Based on the results of the MD simulation, the permeability coefficient of the mixture of components was calculated. The change in the permeability coefficient depending on the thickness of the membrane also characterized its selectivity to water. Since the permeation coefficient should not depend on the membrane thickness, it can be assumed that the results obtained in this work were related to the influence of the transition layer in the membrane at the membrane–solution boundary. Thus, starting from a certain value of membrane thickness, the permeation coefficient should remain constant. Knowledge of this thickness is important for adequate determination of the separation characteristics of polymeric membranes based on MD simulation.

## Figures and Tables

**Table 1:** Simulation results.

**Number of Polymer Layers**	$j_{w}$ **, mole/m^2^s**	$j_{e}$ **, mole/m^2^s**	$S_{w}$	$P_{w}$ $,\;10{-}^{10}\;\frac{mole}{m\;s\;Pa}$	$P_{e}$ $,\;10{-}^{10}\;\frac{mole}{m\;s\;Pa}$
1	2549	4884	0.522	1.46	1.47
2	1431	1793	0.798	1.64	1.08

## Data Availability

The data presented in this study are available in this article. The source of the developed software is released under an open license https://gitflic.ru/project/knrtu/m-dcv.

## Supplementary Materials

The following supporting information can be downloaded at: https://www.mdpi.com/article/10.3390/membranes13020128/s1, Figure S1: Atom names and charges; Table S1: Parameters of the intermolecular interaction potentials.
