# Supplementary material for: Molecular Simulation of Pervaporation on Polyurethane Membranes
Source: Membranes (Basel). 2023 Jan 19;13(2):128. doi: 10.3390/membranes13020128 (PMC9960205; doi:10.3390/membranes13020128)
Supplement: Supplementary file 1 [file membranes-13-00128-s001.zip › membranes-2163498-supplementary.pdf]

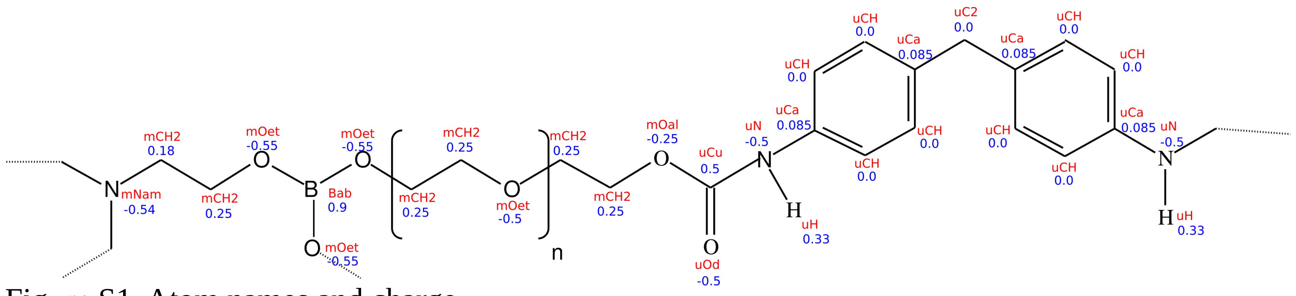

Figure S1. Atom names and charge

Table S1:Parameters of intermolecular interaction

$$\phi_{nb}(r_{ij}) = 4\varepsilon_{ij}\left(\left(\frac{\sigma_{ij}}{r_{ij}}\right)^{12} - \left(\frac{\sigma_{ij}}{r_{ij}}\right)^{6}\right) + \frac{q_i q_j}{4\pi\epsilon_0 r_{ij}}$$

mix rule

$$\varepsilon_{ij} = \sqrt{\varepsilon_i \varepsilon_j} \qquad \sigma_{ij} = \frac{\sigma_i + \sigma_j}{2}$$

Atom type	atomic weight	σ, nm	ε, kJ/mol
AEBA-MDI			
mNam	14.0067	0.378	0.1
mCH2	14.0256	0.395	0.38
mOet	15.999	0.28	0.46
mOal	15.999	0.302	0.77
mHal	1.006	0	0
Bab	10.806	0.36	0.316
uOe	15.999	0.28	0.46
uOd	15.999	0.305	0.66
uCu	12.0107	0.382	0.33
uN	14.0067	0.334	0.92
uH	1.006	0	0
uCa	12.0107	0.388	0.17
uCH	13.0107	0.3695	0.42
uC2	12.0107	0.395	0.38
ethanol			
CE1	15.011	0.375	0.814772
CE2	14.011	0.395	0.382444
OE1	15.9994	0.302	0.773202
HE1	1.008	0.01	0
water			
OW	15.9994	0.31589	0.7749
HW	1.008	0	0

MW	0	0	0

Bond interaction

$$\phi_b(r_{ij}) = \frac{1}{2} k_b (r_{ij} - b)^2$$

Atom i	Atom j	b, nm	k_b, kJ/(mol nm^2)
mCH2	mCH2	0.154	129370
mOet	mCH2	0.141	154700
mOet	Bab	0.1385	100000
mNam	mCH2	0.1448	205000
mCH2	mOal	0.143	154700
mOal	mHal	0.0945	100000
uOe	uCu	0.1327	89540
uCu	uOd	0.1229	238490
uCu	uN	0.1335	205020
uN	uH	0.101	181590
uN	uCa	0.1381	178660
uCa	uCa	0.14	196230
uCa	uCH	0.14	196230
uCH	uCH	0.14	196230
uCa	uC2	0.151	132630
mCH2	uOe	0.143	154700
OW	HW	0.09572	500000
OW	MW	0.01546	500000
CE1	CE2	0.154	500000
CE2	OE1	0.143	500000
OE1	HE1	0.0945	500000

$$\phi_a(r_{ij}) = \frac{1}{2} k_a (r_{ij} - \theta)^2$$

Atom i	Atom j	Atom k	θ, deg	k_a , kJ/(mol rad^2)
mCH2	mNam	mCH2	109.5	235.11
mNam	mCH2	mCH2	109.5	209.17
mCH2	mCH2	mOet	112	208.94
mCH2	mOet	Bab	116	31.33
mOet	Bab	mOet	120	104.47
mCH2	mOal	mHal	109.47	230.13
mCH2	mOet	mCH2	112	250.90

mCH2	mCH2	mOal	109.47	209.36
mCH2	uOe	uCu	115	259.83
mCH2	mCH2	uOe	112	209.83
uOe	uCu	uOd	125	259.83
uOe	uCu	uN	111.4	338.9
uCu	uN	uH	109.5	133.98
uCu	uN	uCa	121.9	209.2
uOd	uCu	uN	122.9	334.72
uN	uCa	uCH	120	292.88
uH	uN	uCa	119.8	146.44
uCa	uCH	uCH	120	263.59
uCH	uCa	uCH	120	263.59
uCH	uCa	uC2	120	292.88
uCa	uC2	uCa	109.5	167.36
HW	OW	HW	104.52	500
MW	OW	HW	52.26	500
CE1	CE2	OE1	109.47	419.0256
CE2	OE1	HE1	108.5	460.5956

Dihedral angles

$$\phi_d(\psi_{ijkl})=\frac{1}{2}\left(C_1(1-\cos(\psi))+C_2(1-\cos(2\psi))+C_3(1+\cos(3\psi))+C_4(1-\cos(4\psi))\right)$$

Atom i	Atom j	Atom k	Atom l	C_1, kJ/mol	C_2, kJ/mol	C_3, kJ/mol	C_4, kJ/mol
mCH2	mNam	mCH2	mCH2	0.24374	-83.30333	203.45749	144.86160
mNam	mCH2	mCH2	mOet	5.51305	2.54779	-1.16342	3.06789
mCH2	mCH2	mOet	Bab	0.00000	0.00000	0.00258	0.00000
mOet	Bab	mOet	mCH2	0.00000	0.57670	0.00000	0.00000
mOet	mCH2	mCH2	mOet	2.09045	0.00000	-1.04523	4.18087
mOet	mCH2	mCH2	mOal	2.09045	0.00000	-1.04523	4.18087
mCH2	mCH2	mOal	mHal	0.00000	0.87159	-0.12117	0.78066
mCH2	mCH2	mOet	mCH2	0.00000	3.01310	-0.68021	2.31876
mOet	mCH2	mCH2	uOe	2.09045	0.00000	-1.04523	4.18087
CE1	CE2	OE1	HE1	3.48889	-0.48504	3.12490	0.00000

$$\phi_d(\psi_{ijkl})=\sum_i C_i(\cos(\psi))^i$$

Atom i	Atom j	Atom k	Atom l	C_0, kJ/mol	C_1, kJ/mol	C_2, kJ/mol	C_3, kJ/mol
uOd	uCu	uN	uCa	25.48	0	-25.48	0
uOd	uCu	uN	uH	20.5	0	-20.5	0
uOe	uCu	uN	uCa	30.29	4.81	-25.48	0

uOe	uCu	uN	uH	20.5	0	-20.5	0
uCu	uN	uCa	uCH	8.79	0	-8.79	0
uH	uN	uCa	uCH	8.79	0	-8.79	0
uN	uCa	uCH	uCH	-0.77	-2.71	4.03	5.97
uCa	uCH	uCH	uCa	30.33	0	-30.33	0
uCH	uCa	uCH	uCH	30.33	0	-30.33	0
uCH	uCH	uCa	uC2	30.33	0	-30.33	0
uCH	uCa	uC2	uCa	0	0	0	0
uOe	uCu	uN	uH	20.5	0	-20.5	0
mCH2	uOe	uCu	uN	16.74	-4.184	-20.92	0
mCH2	uOe	uCu	uOd	21.44	0	-21.44	0
mCH2	mCH2	uOe	uCu	-2.2	-5.2	0.53	0

$$\phi_d(\psi_{ijkl}) = \frac{1}{2} k_d (\psi_0 - \psi)^2$$

Atom i	Atom j	Atom k	Atom l	ψ_0 , deg	k_d, kJ/(mol rad^2)
uCu	uOe	uOd	uN	0	87.86
uCu	uOe	uN	uOd	0	87.86
uN	uCu	uH	uCa	0	8.37
uN	uCu	uCa	uH	0	8.37
uN	uCa	uCH	uCH	0	9.21
uCH	uCH	uCa	uC2	0	9.21
